# An Essential and Synergistic Role of Purinergic Signaling in Guided Migration of Corneal Epithelial Cells in Physiological Electric Fields

**DOI:** 10.33594/000000014

**Published:** 2019-02-28

**Authors:** Ken-ichi Nakajima, Makiko Tatsumi, Min Zhao

**Affiliations:** a Department of Dermatology, University of California at Davis, Sacramento, CA, USA,; b Wakayama Medical University School of Medicine, Wakayama, Japan,; c Department of Ophthalmology, University of California at Davis, Sacramento, CA, USA

**Keywords:** Corneal epithelial cell, Electric field, Galvanotaxis, ATP, Purinergic signaling

## Abstract

**Background/Aims:**

Directional migration of corneal epithelial cells is essential for healing of corneal wounds, which is a robust response mediated by biochemical and bioelectrical cues. Naturally occurring electric fields at corneal wounds provide a powerful guidance cue for directional cell migration, as does extracellular ATP. Our recent large-scale siRNA library screening identified a role for purinergic signaling in the electric field-guided migration (galvanotaxis/electrotaxis) of human corneal epithelial (hTCEpi) cells.

**Methods:**

We examined the effect of extracellular ATP on galvanotaxis of hTCEpi cells. Galvanotactic cell migration was recorded by video microscopy, and directedness and migration speed was calculated. The role of purinergic receptors in galvanotaxis regulation was evaluated by pharmacological inhibition or knocking down of P2X and P2Y receptors.

**Results:**

Addition of ATP enhanced galvanotaxis, and most remarkably sensitized galvanotaxis response to very low level of electric fields in the physiological range (10–30 mV/mm). The stimulatory effect of extracellular ATP was diminished by apyrase treatment. Importantly, cells stimulated with extracellular ATP migrated with significantly increased directedness and speed, which were diminished by knocking down or pharmacological inhibition of P2X and P2Y receptors. Inhibition of pannexin-1 (ATP permeable channel) significantly impaired galvanotaxis. Moreover, pharmacological inhibition of ecto-ATPase enhanced galvanotaxis.

**Conclusion:**

Extracellular ATP and physiological electric fields synergistically enhanced the galvanotaxis response of hTCEpi cells. hTCEpi cells are likely to secrete ATP actively, and purinergic signaling is down-regulated by ecto-ATPases. Both P2X and P2Y receptors coordinately play a role for galvanotaxis of hTCEpi cells.

## Introduction

Many types of cells in our body can sense weak extracellular electric fields (EFs) and migrate toward the cathode or the anode, termed galvanotaxis/electrotaxis [1–4]. Galvanotaxis is involved in various physiological events, such as wound healing, regeneration, angiogenesis, tissue development, and even pathological situations like cancer metastasis [3]. A number of extracellular and intracellular factors have been shown to regulate galvanotaxis responses. For example, a high concentration of extracellular glucose significantly impaired galvanotaxis of corneal epithelial cells [5].

Adenosine-5’-triphosphate (ATP) is well known as an energy source in cells. Every cell in our body continuously generates ATP molecules from various saccharides (mainly glucose) and triglycerides [6–8], and ATP is utilized for many kinds of cellular functions, e.g. movement of motor proteins such as kinesin, dynein, and myosin [9]. ATP is also used as the source of an inorganic phosphate group for kinase reactions. Many kinases in cells catalyze the transfer of a phosphate group from the γ position of the ATP molecule to their substrate molecules such as proteins, nucleic acids, and lipids [10–12]. Numerous recent studies have revealed that cells secrete ATP to the extracellular space, and extracellular ATP molecules can act as signaling molecules [13–16]. Indeed, many physiological functions of cell are regulated by extracellular ATP [13, 14, 17]. The ATP molecule is secreted through large-pore ATP-permeable channels on the plasma membrane. In mammalian cells, pannexin channels are known as one of the ATP-permeable channels [18, 19]. After secretion into the extracellular space, the ATP molecule binds to its receptor on the plasma membrane, termed purinergic receptors, and transduces a signal to the cells [20, 21]. There are three types of purinergic receptors, P1, P2X and P2Y. P1 is a G-protein coupled receptor preferentially activated by adenosine, a nucleoside molecule composed of adenine and ribose [22]. P2X is an ATP-gated non-selective cation channel [23]. Upon binding with ATP, the channel pore will open, and allow cations such as Na^+^ and Ca^2+^ to flow through the pore. The entry of Na^+^ via P2X leads to the depolarization of the plasma membrane, and the activation of various subsequent intracellular processes. P2Y is a G-protein coupled receptor that activates intracellular signaling cascades to regulate various cellular functions [24, 25]. P2Y is activated upon binding with extracellular nucleotides. In addition to ATP, purinergic receptors can be activated by other nucleotides. P2X receptors are more structurally specific than P2Y receptors in agonist selectivity, and P2X receptors are activated principally by ATP, whereas P2Y receptors can be activated by other nucleotides (e.g. UTP) [20, 26]. Interestingly, P2Y_4_, one type of P2Y receptor, is activated by UTP, but is antagonized by ATP in human cells [27]. These purinergic receptors play important roles in the regulation of cellular physiological functions coordinately, and dysregulation of purinergic signaling will cause diseases such as fibrosis, graft-versus-host disease, bone diseases, and cancer [28–31]. In addition, previous studies have shown that ATP concentration is increased at wound sites, suggesting that ATP release may be essential for efficient wound healing [32, 33].

We recently performed large-scale siRNA library screening to identify important genes for galvanotaxis regulation and found that knocking down several genes impaired or enhanced galvanotaxis of corneal epithelial cells [34, 35]. Among the genes we identified, knocking down of P2RX5, one of the P2X receptor-encoding genes, significantly impaired galvanotaxis. In this study, we determined the synergy between purinergic signaling and galvanotaxis in human corneal epithelial cells and the roles of eight P2 receptors in the synergy.

## Materials and Methods

### Materials

EpiLife culture medium with Ca^2+^ (60 μM), EpiLife defined growth supplement (EDGS), penicillin/streptomycin, and Lipofectamine 2000 reagent were purchased from Thermo Fisher Scientific (Waltham, MA USA). On-target plus siRNA was purchased from GE healthcare (Little Chalfont, UK). FNC coating mixture was purchased from Athena ES (Baltimore, MA, USA). Suramin hexasodium salt (an inhibitor of P2X and P2Y receptors), PPADS tetrasodium salt (an inhibitor of P2X and P2Y receptors), TNP-ATP triethylammonium salt (an inhibitor of P2X receptor), NF 157 (an inhibitor of P2Y_11_ and P2X_1_), ARL 67156 (an inhibitor of ecto-ATPases), and ^10^Panx (an inhibitor of Pannexin-1 channel) were purchased from Tocris Bioscience (Minneapolis, MN, USA). Adenosine-5’-(γ-thio)-triphosphate tetralithium salt (ATPγS, a non-hydrolyzable ATP analogue), Brilliant blue G (BBG; an inhibitor of P2X receptors), and apyrase were purchased from Sigma-Aldrich (St. Louis, MO, USA).

### Cell culture and siRNA transfection

Telomerase immortalized human corneal epithelial cells (hTCEpi cells) were generously provided by Dr. James Jester (University of California Irvine), and were grown in EpiLife with 60 μM Ca^2+^ supplemented with EDGS and penicillin/streptomycin at 37 °C with air containing 5% carbon dioxide, as described previously [34]. For siRNA transfection, hTCEpi cells were seeded at the density of 5×10^4^ cells/well in 12-well plate 1 day before transfection. siRNA was transfected by using Lipofectamine 2000 reagent according to the manufacturer’s protocol. At 2 days after transfection, the cells were trypsinized and seeded onto galvanotaxis chamber using FNC coating mixture and subjected to migration experiments described below.

### EF-induced migration

EF-induced cell migration experiments were performed as described previously [34]. Briefly, direct current was applied through agar-salt bridges via Ag/AgCl electrodes in Steinberg’s solution (consisting of 58 mM NaCl, 0.67 mM KCl and 0.44 mM Ca(NO_3_)_2_, 1.3 mM MgSO_4_ and 4.6 mM Tris base, pH 7.4) to pooled medium on either side of the galvanotaxis chamber. Cells were exposed to 0–100 mV/mm direct current EF for 1 h. Cell behavior was observed with a Carl Zeiss Observer Z1 inverted microscope with a Photometrics QuantEM EMCCD camera (Photometrics Inc., Tucson, AZ, USA) and MetaMorph NX software (Molecular Devices, Sunnyvale, CA, USA), and serial time-lapse images were captured. Cell migration was analyzed to determine directedness (cos θ) and track speed by using ImageJ software (NIH, Bethesda, MA, USA) with MTrackJ and Chemotaxis tool plugins. Briefly, trajectories of cells were pooled to make composite graphs. The directedness of migration was assessed as cos θ, where θ is the angle between the EF vector and a straight line connecting start and end positions of a cell. A cell moving directly to cathode would have a directedness of 1; a cell moving directly to the anode would have a directedness of −1. A value close to 0 represents random cell movement. Speed is the total length travelled by the cell divided by time.

### Statistics

All data are represented as means ± SEM. We used Statcel4 plugin (OMS publishing Inc., Tokorozawa, Japan) with Microsoft Excel for statistical analysis. ANOVA, Tukey’s HSD and the Student’s *t*-test were used for statistical analysis as appropriate and a *p* value less than 0.05 was considered as statistically significant.

## Results

### Identification of purinergic receptors through siRNA library screening

We recently performed a large-scale siRNA library screen to identify important molecules for galvanotaxis regulation using human corneal epithelial (hTCEpi) cells. We found that an inwardly rectifying K^+^ channel Kir4.2 (encoded by the KCNJ15 gene) plays a crucial role in sensing weak extracellular EFs in concert with intracellular small molecules (polyamines) to facilitate galvanotaxis [34]. During our siRNA library screening, we found several other molecules whose knocking down significantly impaired or enhanced the galvanotaxis response of hTCEpi cells. Among the molecules we identified in the screening, knocking down of P2RX5 significantly impaired galvanotaxis of hTCEpi cells. P2RX genes encode ligand-gated cation permeable ion channel proteins, and extracellular nucleotide, principally ATP, acts as a ligand to activate P2X receptors.

### Extracellular ATP significantly increased galvanotaxis response in very weak electric fields

**Fig. 1.**
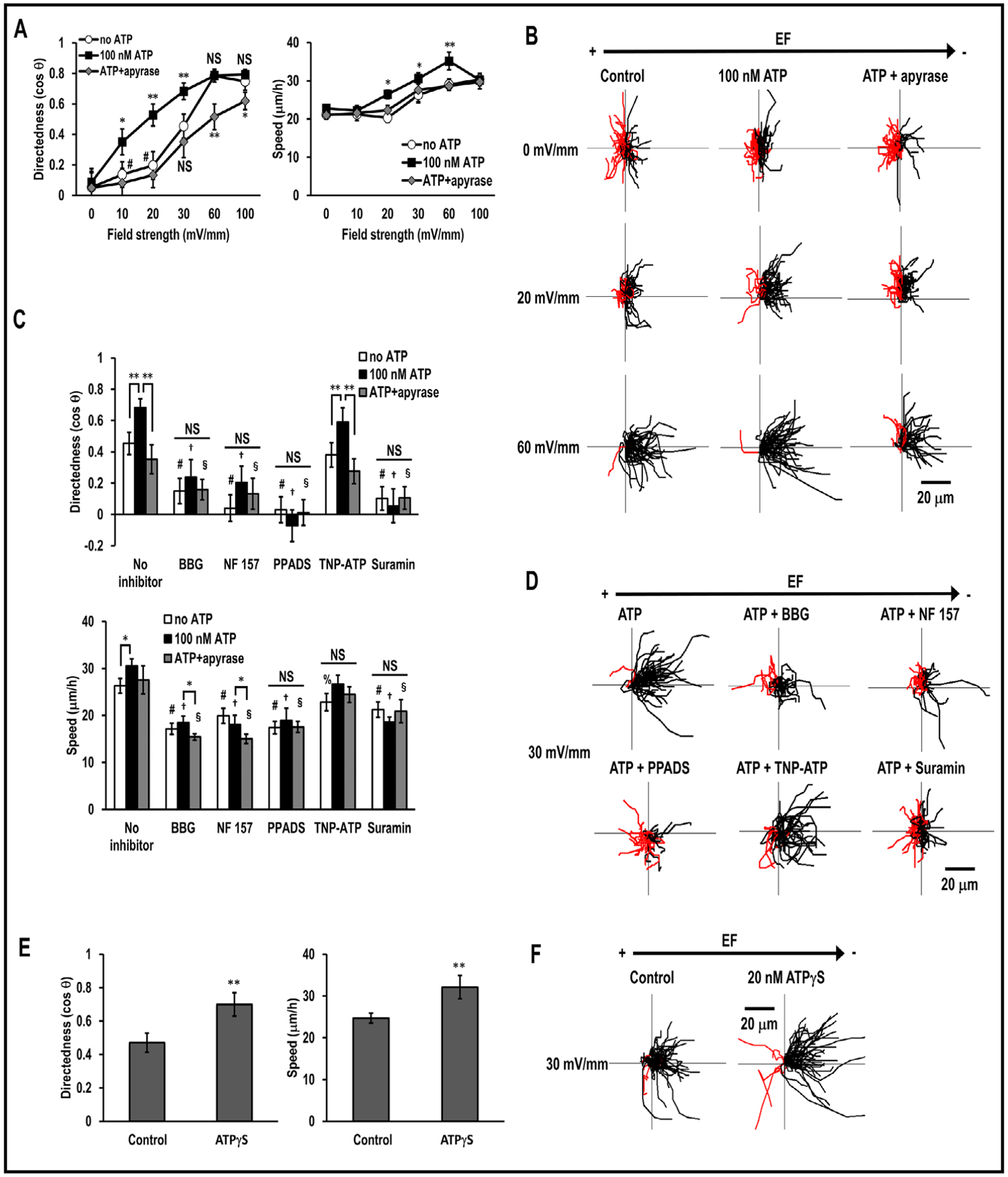
Extracellular ATP sensitized galvanotaxis of hTCEpi cells via purinergic receptors. (A) Galvanotaxis was enhanced in the presence of 100 nM ATP. apyrase (10 U/ml) diminished the stimulatory effect of ATP. Data represented as mean ± SEM. *p<0.05, **p< 0.01 vs. no ATP. Note that there are no statistically significant differences between 0 mV/mm, 10 mV/mm, and 20 mV/mm of no ATP groups (#). (B) Migration trajectories in the presence or absence of ATP and apyrase. Black and red lines indicate trajectories of cells that migrated toward the cathode (right side) and the anode (left side), respectively. (C) Cells treated with inhibitors of purinergic receptors lost galvanotaxis (5 μM BBG, 3 μM NF 157, 30 μM PPADS, 10 μM TNP-ATP, or 100 μM suramin in the presence or absence of 100 nM ATP and 10 U/ml apyrase). EF=30 mV/mm. Data represented as mean ± SEM. *p<0.05, and **p< 0.01 between two groups indicated in the figure. #p<0.01 vs. no inhibitor no ATP group. †p<0.01 vs. no inhibitor 100 nM ATP group. §p<0.01 vs. no inhibitor ATP+apyrase group. %p<0.05 vs. no inhibitor no ATP group. (D) Migration trajectories in the presence or absence of inhibitors. (E) Non-hydrolyzable ATP analogue ATPμS (20 nM) enhanced galvanotaxis. EF=30 mV/mm EF. Data represented as mean ± SEM. **p< 0.01. (F) Migration trajectories in the presence or absence of ATPμS. n=40–50 for each group.

Recent studies have revealed that many types of cells in our body secrete ATP, and secreted extracellular ATP regulates various cellular functions via binding with P2 receptors [13–17]. This autocrine/paracrine ATP signaling system plays fundamental roles in regulating cellular functions, including cell proliferation and metabolism. It is also suggested that wounding increases tissue ATP concentration [32]. Of note, purinergic signaling is implicated in wound healing in corneal epithelium [32, 36–38]. The increase in ATP concentration after wounding is generally assumed to result from cytoplasmic leakage from damaged cells (passive leakage). In addition, surrounding cells and immune cells at wound sites actively secrete ATP. The passively leaked and actively secreted ATP in the wound site enhances cell proliferation, migration and angiogenesis to promote efficient tissue repair. Epithelial tissues possess a unique electrical property termed transepithelial potential difference (TEP) [39–41]. When an epithelial layer is damaged, the TEP will drive electrical current flow, and generate a wound electric field (EF). This electrical current/EF induces directional migration of epithelial cells toward the wound to facilitate wound closure [42, 43]. Indeed, inhibition of galvanotaxis significantly delayed wound healing [44]. As mentioned above, we found that knocking down of P2RX5 impaired galvanotaxis of hTCEpi cells through our previous siRNA library screening. We hypothesized that extracellular ATP enhances galvanotaxis of hTCEpi cells through binding with purinergic receptor(s). In order to test this hypothesis, we first applied ATP (100 nM) to hTCEpi cells and evaluated galvanotactic migration of the cells. As shown in Fig. 1A and B, application of 100 nM ATP significantly enhanced the galvanotaxis response of hTCEpi cells. In control condition (without addition of ATP), directedness (cos θ value) at the EF strength of 10 mV/mm and 20 mV/mm was not statistically different from 0 mV/mm group (indicated by #). The cells started to respond significantly to directionally migrate at the EF strength of 30 mV/mm without addition of ATP (open circles in Fig. 1A). In contrast, in the presence of 100 nM ATP, the cells started to respond at 10 mV/mm. Directedness value at 10 mV/mm with ATP is significantly higher than that at 0 mV/mm. In addition, directedness values of the ATP-treated group at the EF strength of 10, 20 and 30 mV/mm were significantly higher than those of the control group (closed squares in Fig. 1A). There was no difference between the control and ATP-treated group at the EF strength of 60 mV/mm or higher, suggesting that the effect of ATP application may be overridden by other factors. Next we tested if extracellular ATP really stimulated galvanotaxis of hTCEpi cells. To do this we added apyrase, an enzyme catalyzing hydrolysis of ATP to AMP and inorganic phosphates, to degrade extracellular ATP. As shown in Fig. 1A and B, the stimulatory effect of ATP was completely abolished by addition of apyrase (see gray diamonds in Fig. 1A). Moreover, directedness values of the ATP+apyrase group were significantly lower than those of control (no ATP) group at 60 mV/mm and 100 mV/mm.

If the effect of ATP application were mediated through purinergic receptors, the effect of ATP would be abolished by blockage of these receptors. In order to assess the contribution of purinergic receptors, we next applied inhibitors of purinergic receptors (both P2X and P2Y receptors). Suramin and PPADS, both non-specific inhibitors of several P2X and P2Y receptors, abolished the effect of extracellular ATP application. BBG, an inhibitor of several P2X receptors, and NF 157, an inhibitor of P2Y_11_ and P2X_1_, also significantly attenuated the stimulatory effect of ATP. However, another P2X inhibitor TNP-ATP had little effect on galvanotaxis of hTCEpi cells (Fig. 1C and D).

Of note, in addition to ATP, a non-hydrolysable analogue of ATP, ATPγS, also significantly enhanced galvanotaxis of hTCEpi cells (Fig. 1E and F). In the presence of ATPγS, both directedness and migration speed were significantly higher than those of the control group. Taken together, these results show that extracellular ATP enhanced galvanotaxis via P2X and P2Y receptors in hTCEpi cells. Activation of purinergic signaling by ATP thus sensitizes the galvanotaxis reaction of human corneal epithelial cells.

### Blocking pannexin-1 channel significantly reduced ecto-ATPase enhanced galvanotaxis

**Fig. 2.**
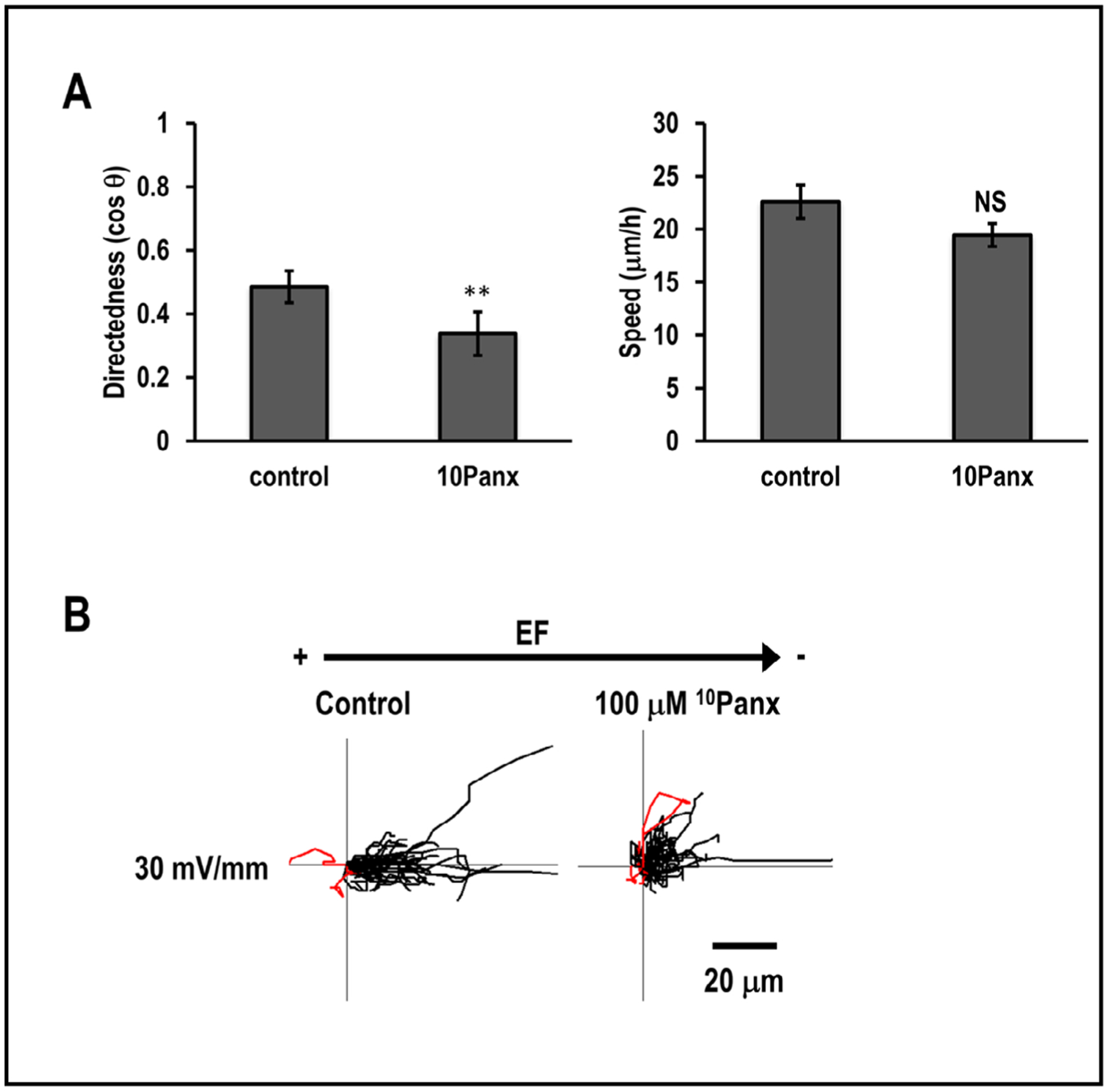
Inhibition of pannexin-1 ATP-permeable channel significantly impaired galvanotaxis of hTCEpi cells. (A) Directedness and migration speed of cells treated with 100 μM of ^10^Panx at the EF strength of 30 mV/mm. (B) Black and red lines indicate trajectories of cells that migrated toward the cathode and the anode side, respectively. n=50 cells for each group. Statistical analysis was performed. Data represented as mean ± SEM. **p< 0.01.

Pannexin channels act as the ATP secreting channels in mammalian cells [18]. Secreted ATP through the ATP-permeable channels activates cells by binding with purinergic receptors. This active ATP secretion (as well as passive leakage) and autocrine/paracrine ATP signaling regulates various cellular functions. Thus, we blocked pannexin-1 channel by using its specific inhibitor ^10^Panx and evaluated its effect on galvanotaxis in hTCEpi cells. As shown in Fig. 2, application of ^10^Panx slightly but significantly lowered directedness (cos θ) at the EF strength of 30 mV/mm. This result suggests that hTCEpi cells actively secrete ATP through pannexin-1 channel.

**Fig. 3.**
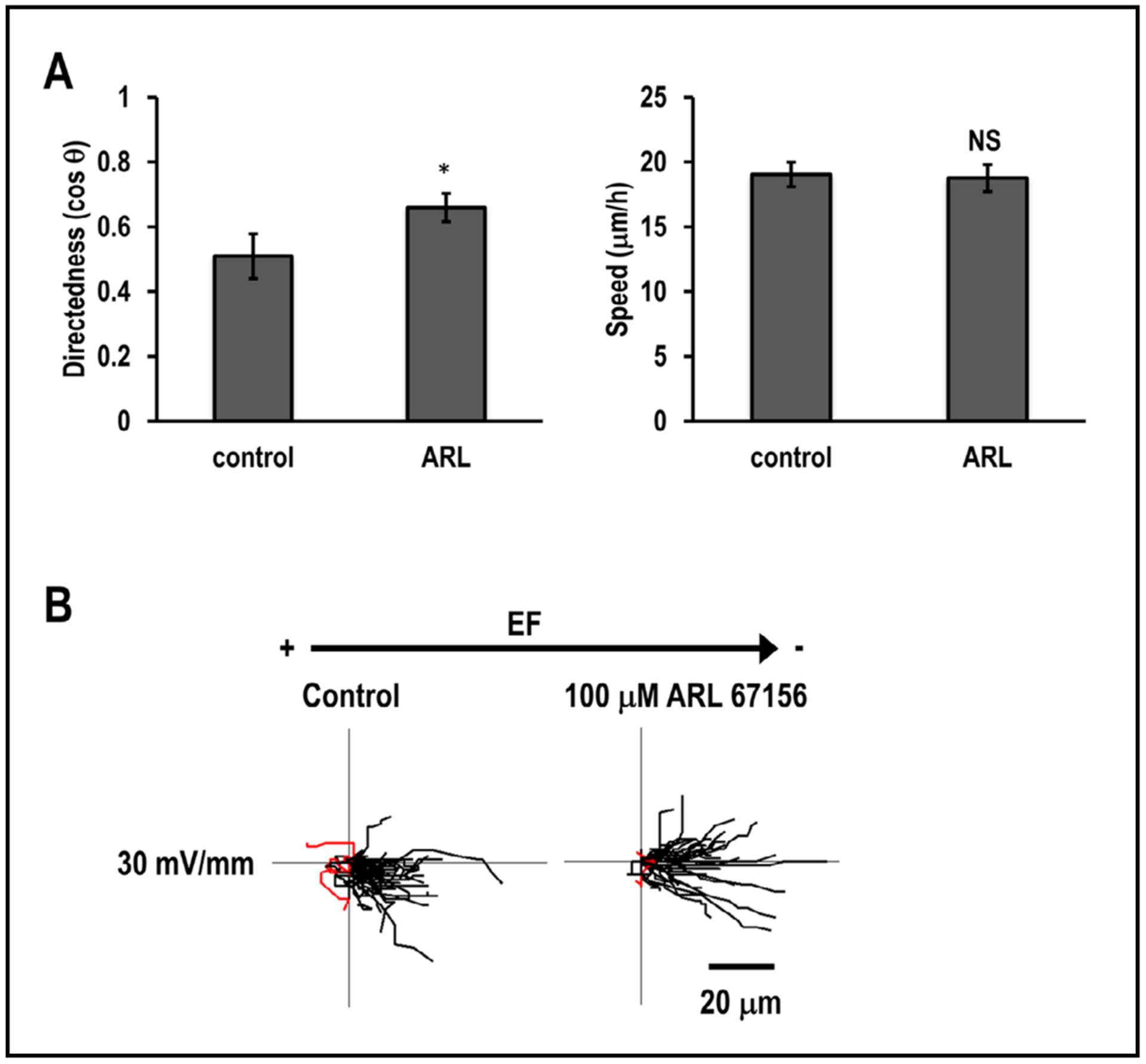
Inhibition of ecto-ATPases significantly enhanced galvanotaxis of hTCEpi cells. (A) Directedness and migration speed of cells treated with 100 μM of ARL 67156 at the EF strength of 30 mV/mm. (B) Black and red lines indicate trajectories of cells that migrated toward the cathode and the anode side, respectively. n=50 cells for each group. Statistical analysis was performed. Data represented as mean ± SEM. *p< 0.05.

Extracellular ATP is catabolized by a variety of extracellular ATPases and nucleotidases. These ATPases and nucleotidases, termed ecto-ATPases and ectonucleotidases, are variably expressed on the cell surface of various cell types [45–47]. It has been suggested that ecto-ATPases are essential for terminating ATP-mediated signaling through purinergic receptors. If ecto-ATPases in corneal epithelial cells play such a role, inhibition of ecto-ATPases would enhance the galvanotaxis reaction of hTCEpi cells. To test this possibility, we next applied an ecto-ATPase inhibitor ARL 67156 and evaluated the effect on galvanotaxis. As shown in Fig. 3, application of ARL 67156 significantly increased the galvanotaxis reaction of hTCEpi cells at the EF strength of 30 mV/mm. Taken together, these results show that hTCEpi cells actively secrete ATP molecules, and ecto-ATPases degrade ATP to negatively regulate purinergic signaling for galvanotaxis. These results suggest that ATP is secreted via pannexin-1 channel, and purinergic signaling is negatively regulated by ecto-ATPases.

### Inhibition of either P2X and P2Y receptors inhibited the effects of ATP sensitization

**Fig. 4.**
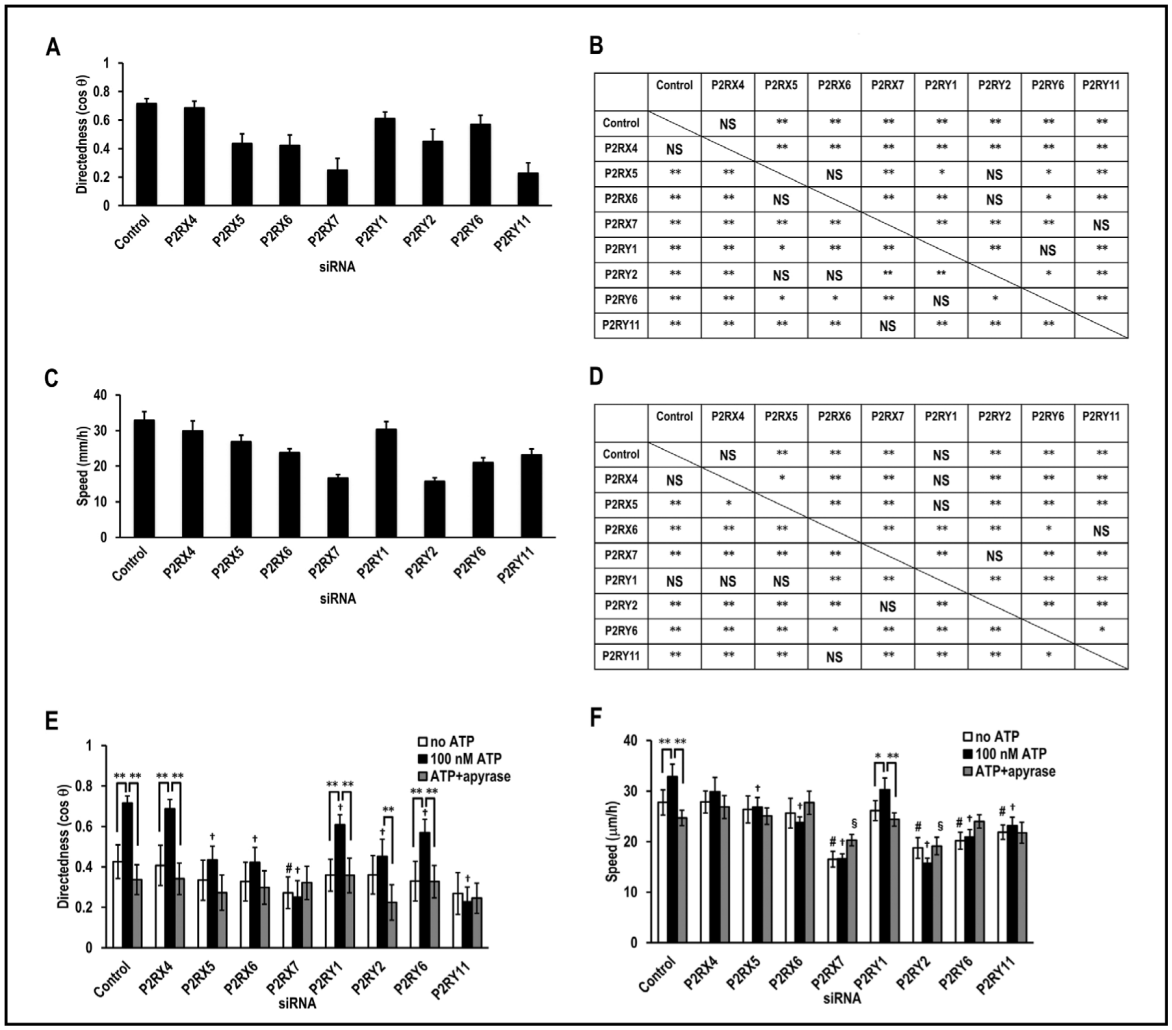
Knocking down of purinergic receptors diminished the stimulatory effect of ATP. siRNA against P2RX and P2RY was individually transfected into hTCEpi cells using Lipofectamine 2000 reagent. Cell migration was recorded in the presence of 100 nM ATP at the EF strength of 30 mV/mm, and directedness (A) and migration speed (C) was calculated. n=50 cells for each group. Results confirmed in two separate experiments. Statistical analysis was performed. Data represented as mean ± SEM. n=50 for each group. Statistical analyses were performed between each group, for directedness (B) and for migration speed (D). *p<0.05, **p< 0.01, and NS not significant between two groups. (E and F) Apyrase treatment attenuated the effect of ATP. siRNA against P2X and P2Y was individually transfected into hTCEpi cells by using Lipofectamine 2000 reagent. Cell migration was recorded in the presence or absence of 100 nM ATP and 10 U/ml apyrase at the EF strength of 30 mV/mm, and directedness (E) and migration speed (F) was calculated. n=40–50 cells for each group. Statistical analysis was performed. Data represented as mean ± SEM. *p<0.05, and **p< 0.01 between two groups indicated in the figure. #p<0.01 vs. Control siRNA no ATP group (leftmost column). †p<0.01 vs. Control siRNA 100 nM ATP group (second column from the left). §p<0.01 vs. Control siRNA ATP+apyrase group (third column from the left).

In purinergic signaling, extracellular ATP binds with its receptor molecules on the plasma membrane, purinergic P2X and P2Y receptors. As mentioned above, P2X receptors are ligand-gated cation channels. P2Y receptors are G-protein coupled receptor (GPCR). Seven P2X receptor-encoding genes (P2RX1, P2RX2, P2RX3, P2RX4, P2RX5, P2RX6, and P2RX7), and ten P2Y receptor-encoding genes (P2RY1, P2RY2, P2RY4, P2RY6, P2RY8, P2RY10, P2RY11, P2RY12, P2RY13, P2RY14,) have been identified in the human genome. Four P2X receptor-encoding genes (P2RX4, P2RX5, P2RX6, and P2RX7) and four P2Y receptor-encoding genes (P2RY1, P2RY2, P2RY6, and P2RY11) are expressed in hTCEpi cells, at least at the mRNA level (Nakajima, unpublished data). We knocked down all P2 receptors expressed in hTCEpi cells individually by siRNA. We transfected siRNA into hTCEpi cells using Lipofectamine 2000. At 2 days after transfection, galvanotaxis of those cells were evaluated at the EF strength of 30 mV/mm in the presence or absence of ATP. As shown in Fig. 4A and B, knock down of three P2X-encoding genes (P2RX5, P2RX6, and P2RX7) and all P2Y-encoding genes (P2RY1, P2RY2, P2RY6, and P2RY11) tested in this study significantly lowered directedness (cos θ value) of galvanotaxis of hTCEpi cells at the EF strength of 30 mV/mm in the presence of ATP. Knock down of those P2 receptor-encoding genes also slowed down of migration speed of the cells (Fig. 4C and D). Knocking down those P2 receptors also slightly inhibited the galvanotaxis reaction of hTCEpi cells in the absence of ATP (Fig. 4E and F). Importantly, addition of apyrase attenuated the stimulatory effect of ATP (Fig. 4E and F), indicating that extracellular ATP enhances galvanotaxis of hTCEpi cells via three P2X receptors (P2X_5_, P2X_6_, and P2X_7_) and four P2Y receptors (P2Y_1_, P2Y_2_, P2Y_6_, and P2Y_11_) expressed in these cells.

## Discussion

In the present study, we demonstrated that extracellular ATP significantly lowered threshold EF strength for the galvanotaxis response in hTCEpi cells via purinergic receptors. Recent studies have revealed that purinergic signaling is one of key factors in corneal wound healing [37]. For example, Yin et al. reported that wound-induced ATP release triggers EGFR activation [32]. In addition, purinergic receptors are expressed in corneal epithelial cells, and purinergic signaling is required for corneal reepithelialization during the wound healing process [36, 38]. Our recent siRNA library screening determined that a purinergic receptor plays a role for galvanotaxis in hTCEpi cells [34]. We thus explored the physiological significance of purinergic receptors and extracellular ATP for galvanotaxis regulation. Many type of epithelial tissues have unique electrical properties, called transepithelial potential difference (TEP). This potential difference is established by an uneven distribution of cations and anions between epithelial layer(s) [39–41]. When epithelial tissue is damaged, the potential difference will drive electrical current flow and also generate an EF. The wound center is more negative than the distal regions, so current will flow laterally from distal areas to the wound. It has been suggested that, in the physiological situation, the strength of EF around the wound is 10–100 mV/mm, depending on basal TEP, which is established and maintained by the ion-transporting system in the epithelial cells (e.g. Na^+^/K^+^ ATPase), and wound size and depth [48]. Wound-generated electrical current/EF plays pivotal roles for repair of the tissue efficiently, since most types of epithelial cells can sense and respond robustly to the electrical current/EF and migrate in a certain direction, in this case toward the cathode, which is the center of the wound. In the present study, we demonstrated that hTCEpi cells showed significant directional migration toward the cathode at the EF strength of 30 mV/mm, but not at the EF strength of 10 or 20 mV/mm without addition of ATP. In the presence of extracellular 100 nM ATP, hTCEpi cells showed significant directional migration at the EF strength of 10 mV/mm and 20 mV/mm, and at the EF strength of 10 to 30 mV/mm the cells migrated more directionally than control (no ATP added) group. Addition of apyrase, an enzyme that catalyzes hydrolysis of ATP to AMP and inorganic phosphates, completely abolished the stimulatory effect of ATP addition. This result clearly indicates that extracellular ATP sensitizes the galvanotaxis reaction of hTCEpi cells. As mentioned above, an epithelial wound generates approximately 10–100 mV/mm EF. Our results indicate that extracellular ATP lowered the threshold EF strength for galvanotaxis of corneal epithelial cells for efficient wound healing. Application of apyrase with ATP significantly lowered directedness values at high EF strength (60 mV/mm and 100 mV/mm) compared to no ATP group (Fig. 1A), suggesting that extracellular ATP may play some roles at higher EF, in addition to the role at lower EF. ATP secretion in wound lesion, by active ATP secretion from surrounding intact cells and passive cytoplasmic ATP leakage from damaged cell, is thought to contribute for tissue repair [32]. Our results suggest that extracellular ATP (from both active secretion and passive leakage) contributes to tissue repair after wounding by facilitation of directional cell migration in response to wound-generate weak electrical current/EF. Synergistic action of extracellular ATP and EF might be important for efficient wound healing and tissue repair.

Importantly, the effect of extracellular ATP application was diminished by treatment with various inhibitors of P2 receptors. We used suramin (a non-specific inhibitor of both P2X and P2Y receptors), PPADS (a non-specific inhibitor of both P2X and P2Y receptors), TNP-ATP (an inhibitor of P2X receptors), BBG (an inhibitor of P2X receptors), and NF 157 (an inhibitor of P2Y_11_ and P2X_1_). The effect of extracellular ATP application was attenuated by treatment with suramin, PPADS, BBG, and NF 157, but not with TNP-ATP. Those inhibitors have different specificity against P2X or P2Y receptors, or both P2X and P2Y receptors. For example, suramin inhibits several P2X and P2Y receptors (P2X_2_, P2X_5_, P2Y_2_, P2Y_4_, and P2Y_11_), whereas TNP-ATP inhibits several P2X receptors (P2X_1_, P2X_3_, and P2X_4_). As mentioned above, four P2X receptors-encoding genes (P2RX4 to P2RX7) and four P2Y receptor-encoding genes (P2RY1, P2RY2, P2RY6, P2RY11) are expressed in hTCEpi cells (Nakajima, unpublished results). To evaluate which P2 receptors are functionally expressed and contribute to galvanotaxis in hTCEpi cells, we knocked down those four P2X-encoding genes and four P2Y-encoding genes individually by using siRNA. As shown in Fig. 4, knocking down three P2X-encoding genes (P2RX5, P2RX6, and P2RX7) and four P2Y-encoding genes (P2RY1, P2RY2, P2RY6, P2RY11) significantly decreased directedness (cos θ value), and knock down of some of them also reduced migration speed in the presence of ATP. This result suggests that three P2X (P2X_5_, P2X_6_, and P2X_7_) and four P2Y receptors (P2Y_1_, P2Y_2_, P2Y_6_, and P2Y_11_) are functionally expressed and contribute to galvanotaxis of hTCEpi cells. Consistent with the results of inhibitor experiments shown in Fig. 1C, P2X_4_ seems to play a small role in the galvanotaxis reaction of hTCEpi cells, since knocking down of P2RX4 and treatment with TNP-ATP, an inhibitor of P2X_1_, P2X_3_, and P2X_4_, had little effect on galvanotaxis. As mentioned above, multiple P2X and P2Y receptors are expressed in hTCEpi cells. Therefore, knocking down of one receptor may cause compensatory action by other receptor(s). In the present study, knocking down of some receptors (except for P2X_4_) significantly impaired galvanotaxis, but the effect was not so strong as that of some inhibitors such as PPADS and suramin, which inhibit multiple receptors, suggesting possible compensatory effects. Knocking down of one receptor, for example P2X_5_, may up-regulate P2X_6_/P2X_7_ expression and/or activity, and up-regulated P2X_6_/P2X_7_ may compensate the loss of P2X_5_. This might be the reason why the inhibitory effect by knocking down of several receptors was not so strong as with acute drug treatment, despite their knocking down significantly impairing galvanotaxis.

P2X receptors are ligand-gated non-selective cation channels that have permeability toward cations such as Na^+^, K^+^ and Ca^2+^. How does extracellular ATP enhance the galvanotaxis reaction through binding with P2X receptors? One possible explanation would be membrane potential change. A number of studies have shown that membrane potential is one of the key factor for galvanotaxis response of various cell types. For example, Gao et al. reported that reduced membrane potential in *Dictyostelium* significantly impaired galvanotaxis without any significant effect on chemotactic motility [49]. Moreover, our recent study showed that inhibition of inwardly rectifying K^+^ channel KCNJ15/Kir4.2 significantly depolarized membrane potential of hTCEpi cells, and this might be a reason for impaired galvanotaxis upon KCNJ15/Kir4.2 inhibition/knockdown [34]. Furthermore, Zhou et al. recently demonstrated that membrane depolarization influenced the organization of plasma membrane phospholipids, and this in turn regulated localization and activity of the small GTP-binding protein K-Ras [50]. It is well known that K-Ras regulates many cellular functions, including cell migration [51]. Castellano et al. showed that Ras interacts with the p110α subunit of phosphatidylinositol-3-kinase, and regulates cell motility [51]. Phosphatidylinositol-3-kinase and its product phosphatidylinositol-3, 4,5-trisphosphate (PIP_3_) have been shown to play pivotal roles in galvanotaxis regulation [34, 52]. In the present study, activation of P2X receptors may regulate directional cell migration via similar mechanisms. On the other hand, P2Y receptors are the G-protein coupled receptors (GPCRs) that couple with heterotrimeric G-proteins and transduce signals to downstream signaling molecules, such as adenylate cyclase and phospholipase C (PLC) [53]. Those downstream signaling molecules regulate a variety of cellular functions [54–56]. For example, diacylglycerol (DAG) and inositol-3, 4,5-trisphosphate (IP_3_) is generated upon PLC activation, and IP_3_ triggers Ca^2+^ release from endoplasmic reticulum. Both DAG and Ca^2+^ (released from endoplasmic reticulum) activate the MEK-ERK pathway to enhance cell migration [57]. Elucidation of the detailed molecular mechanisms of how ATP activates downstream pathways of P2X and P2Y receptors and their contribution to galvanotaxis is an important future issue.

All P2X receptors are principally activated by ATP, while some P2Y receptors are activated by other nucleotide(s), such as ADP, UTP, UDP, and UDP-glucose [20, 26]. Interestingly, ATP antagonizes P2Y_4_ [27]. In hTCEpi cells, four P2Xs (P2X_4_, P2X_5_, P2X_6_, and P2X_7_) and four P2Ys (P2Y_1_, P2Y_2_, P2Y_6_, and P2Y_11_) are expressed, and all of those P2 receptors except P2X_4_ play a role in galvanotaxis regulation (Fig. 4). Among the P2 receptors functionally expressed in hTCEpi cells, P2Y_6_ is strongly activated by UDP and UTP rather than ATP [58]. In the present study, knocking down P2RY6 significantly impaired galvanotaxis. These results suggest that hTCEpi cells may secrete other nucleotides, such as UTP and UDP, in addition to ATP, and those uridine nucleotides may also stimulate galvanotaxis via the P2Y_6_ receptor. Further studies will be necessary to clarify functional contributions of different type of nucleotides in galvanotaxis regulation comprehensively.

Application of ^10^Panx, an inhibitor of the pannexin-1 channel, significantly impaired galvanotaxis of hTCEpi cells. On the other hand, application of ARL 67156, an inhibitor of ecto-ATPases, significantly enhanced galvanotaxis. These results suggest that hTCEpi cells secrete ATP via a pannexin-1 ATP permeable channel, and this “autocrine/paracrine purinergic signaling” is negatively regulated by ecto-ATPases. Active ATP secretion and autocrine/paracrine ATP signaling is thought to be important for the maintenance of homeostasis in various tissues, including epithelia. Three pannexin channel-encoding genes have been identified in the human genome, namely Panx1, Panx2, and Panx3. Pannexin-1 is encoded by the Panx1 gene and expressed ubiquitously. Pannexin channels can be activated by various factors, such as mechanical stimulation, caspase cleavage, cytosolic Ca^2+^, extracellular ATP and K^+^, and membrane depolarization [59–64]. Exposure to an EF alters membrane potential; the cathode-facing side becomes more depolarized while the anode-facing side becomes more hyperpolarized [65]. In the present study, exposure to EF may activate the pannexin-1 channel through membrane potential change and cause ATP release. Further studies will be necessary to reveal the activation mechanism of pannexin in response to extracellular EF. Ecto-ATPases (also known as NTPDases) are enzymes that hydrolyze extracellular nucleotides, including ATP and UTP. Eight ecto-ATPase isoforms have been identified so far. Ecto-ATPases degrade extracellular nucleotides to reduce concentration of the ligands of P2 receptors for the down-regulation of P2 receptor-mediated signaling [45–47]. ATP (and other nucleotide triphosphates) is hydrolyzed to ADP, and then to AMP by ecto-ATPases. Finally AMP is degraded to adenosine and inorganic phosphate by ecto-5’-nucleotidase/CD73 [66]. Adenosine is a ligand of another type of purinergic receptor termed P1 receptors [22]. Similar to P2Y receptors, P1 receptors couple with heterotrimeric G-protein and activate or inhibit downstream signaling pathways such as adenylate cyclase and PLC. It is interesting to question what is the role of P1 receptors in galvanotaxis regulation, and what are the unique roles (or the common roles) of P1 and P2Y receptors in galvanotaxis regulation.

## Conclusion

**Fig. 5.**
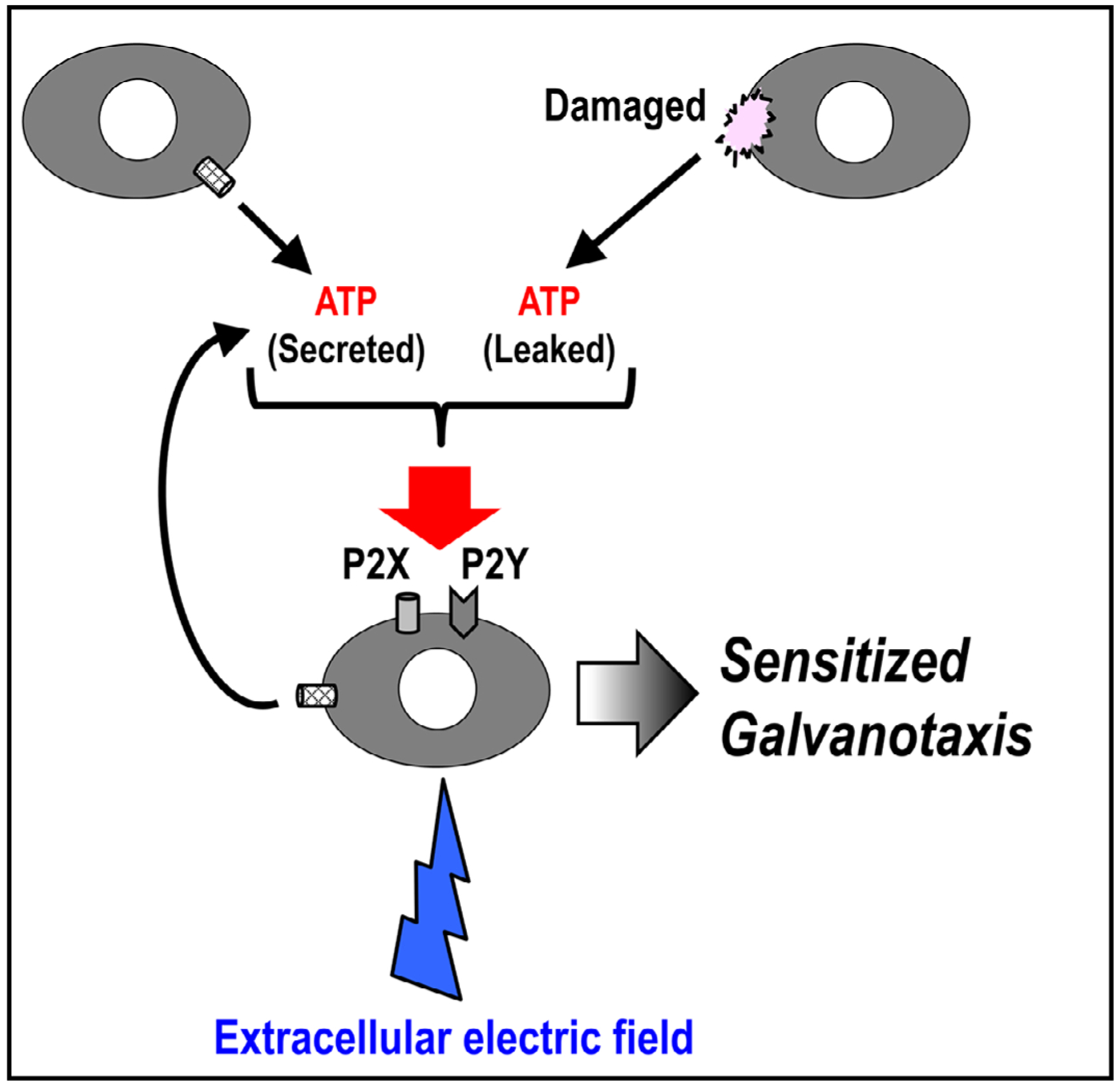
Proposed model for the synergistic role of extracellular ATP and EF for galvanotaxis regulation. Two sources of ATP, passive leakage from damaged cells and active secretion from neighboring cells, and extracellular physiological EF synergistically regulate galvanotaxis of corneal epithelial cells.

We found that extracellular ATP and physiological EF synergistically induced the galvanotaxis response of corneal epithelial cells. Corneal epithelial cells are likely to secrete ATP actively, and purinergic signaling is down-regulated by ecto-ATPases. Both P2X and P2Y receptors coordinately play a role for galvanotaxis of hTCEpi cells. Our proposed model is depicted in Fig. 5. Both actively secreted and passively leaked ATP may help corneal epithelial cells to migrate efficiently by lowering the threshold EF strength during the wound healing process.
